# Downregulation of MHC Class I Expression by Influenza A and B Viruses

**DOI:** 10.3389/fimmu.2019.01158

**Published:** 2019-05-29

**Authors:** Marios Koutsakos, Hamish E. G. McWilliam, Turgut E. Aktepe, Svenja Fritzlar, Patricia T. Illing, Nicole A. Mifsud, Anthony W. Purcell, Steve Rockman, Patrick C. Reading, Julian P. Vivian, Jamie Rossjohn, Andrew G. Brooks, Jason M. Mackenzie, Justine D. Mintern, Jose A. Villadangos, Thi H. O. Nguyen, Katherine Kedzierska

**Affiliations:** ^1^Department of Microbiology and Immunology, Peter Doherty Institute for Infection and Immunity, The University of Melbourne, Parkville, VIC, Australia; ^2^Department of Biochemistry and Molecular Biology, Bio21 Molecular Science and Biotechnology Institute, The University of Melbourne, Parkville, VIC, Australia; ^3^Infection and Immunity Program, Department of Biochemistry and Molecular Biology, Biomedicine Discovery Institute, Monash University, Clayton, VIC, Australia; ^4^Seqirus, Parkville, VIC, Australia; ^5^World Health Organisation (WHO) Collaborating Centre for Reference and Research on Influenza, Peter Doherty Institute for Infection and Immunity, Melbourne, VIC, Australia; ^6^Infection and Immunity Program, Department of Biochemistry and Molecular Biology, Biomedicine Discovery Institute, Monash University, Clayton, VIC, Australia; ^7^Australian Research Council Centre of Excellence for Advanced Molecular Imaging, Monash University, Clayton, VIC, Australia; ^8^Institute of Infection and Immunity, Cardiff University School of Medicine, Heath Park, Cardiff, United Kingdom

**Keywords:** influenza A virus, influenza B virus, MHC-I, HLA, T cells

## Abstract

Manipulation of the MHC-I presentation pathway, and thus limiting MHC-I cell surface expression, is used by many viruses to evade immune recognition. In particular, downregulation of MHC-I molecules at the cell surface can reduce the ability of CD8^+^ T cells to recognize viral peptides presented by MHC-I molecules and thereby delay viral clearance by CD8^+^ T cells. To date, MHC-I downregulation by influenza viruses has not been reported. Given that influenza virus infections are a global health concern and that CD8^+^ T cells play an important role in promoting influenza virus clearance and recovery from influenza disease, we investigated whether influenza A and B viruses (IAV, IBV) downregulated MHC-I as a novel mechanism to evade cellular immunity. Here, we showed that infection of several cell types, including epithelial A549 cells, with a panel of IAV and IBV viruses downregulated the surface MHC-I expression on IAV/IBV-infected cells during the late stages of influenza virus infection *in vitro*. This observation was consistent across a panel of class I-reduced (C1R) cell lines expressing 14 different HLA-A or -B alleles and a panel of 721.221 cell lines expressing 11 HLA-C alleles. Interestingly, IBV infection caused more pronounced reduction in surface MHC-I expression compared to IAV. Importantly, the two viruses utilized two distinct mechanisms for MHC-I downregulation. Our data demonstrated that while IAV caused a global loss of MHC-I within influenza-infected cells, IBV infection resulted in the preferential loss of MHC-I molecules from the cell surface, consequent of delayed MHC-I trafficking to the cell surface, resulting from retaining MHC-I intracellularly during IBV infection. Overall, our study suggests that influenza viruses across both IAV and IBV subtypes have the potential to downregulate MHC-I surface expression levels. Our findings provide new insights into the host-pathogen interaction of influenza A and B viruses and inform the design of novel vaccine strategies against influenza viruses.

## Introduction

Influenza A and B viruses (IAV and IBV, respectively) circulate annually during seasonal epidemics causing significant morbidity and mortality. Two subtypes of IAV (H1N1 and H3N2) and two antigenically distinct lineages of IBV (B-Victoria and B-Yamagata) co-circulate in every season (1, 2). Protection against influenza viruses can be mediated by strain-specific or rare broadly cross-reactive antibodies, as well as broadly cross-reactive CD8^+^ T cells (2, 3). Cytotoxic CD8^+^ T cells provide protection by recognizing viral peptides loaded onto MHC-I molecules on the cell surface of infected cells, which triggers the delivery of cytotoxic molecules and releasing antiviral cytokines to kill the infected cells (4, 5). In this way, CD8^+^ T cells promote viral clearance and recovery during seasonal as well as pandemic and avian influenza virus infection, which share internally conserved peptide sequences (6–10). As a result, and due to their ability to cross-react across highly divergent influenza strains, CD8^+^ T cells are considered an important target for designing universal influenza vaccines that do not require annual reformulation.

The key determinant of CD8^+^ T cell recognition of virally-infected cells is the engagement of the T cell receptor (TCR) to its cognate peptide/MHC-I complex. As MHC-I traffics through the endoplasmic reticulum (ER), short peptides (7–13 amino acids in length) derived from host or foreign proteins are loaded onto MHC-I and then presented on the cell surface. Thus, due to the crucial role of MHC-I in CD8^+^ T cell recognition of the infected cells, many viruses such as HIV and herpesviruses have evolved intricate mechanisms to interfere with MHC-I presentation of viral peptides (11, 12). Such escape mechanisms range from inhibiting the proteasome or the delivery of peptides to the ER for loading onto MHC-I to active retention of MHC-I in intracellular compartments and/or increased rates of internalization and degradation of MHC-I. While such virus-mediated downregulation of MHC-I expression has been shown for a number of viruses (11, 12), downregulation of MHC-I by influenza viruses has not been reported thus far.

By examining the expression levels of MHC-I following IAV or IBV infections, we show that both viruses reduce expression of MHC-I in the late stages of infection *in vitro*, albeit via two different mechanisms. While IAV causes degradation of total MHC-I in the infected cells, IBV causes retention of MHC-I in an intracellular compartment. Interestingly, this seems to be a non-specific effect of the viral life cycle as other surface host protein are downregulated equally, and thus may not have specifically evolved to evade the CD8^+^ T cell response. Overall, our study reveals that IAV and IBV are capable of downregulating MHC-I expression. Understanding the exact mechanisms underlying MHC-I downregulation by influenza viruses and the impact on anti-influenza T cell responses will inform the rational design of new vaccine strategies.

## Materials and Methods

### Cell Lines and Reagents

C1R cells or 721.221 cells (stable-transfectants expressing specific HLA alleles) (University of Melbourne) and THP-1 cells (ATCC) were propagated in RF10 media [RPMI-1640 with 10% heat-inactivated FCS, 1 mM MEM sodium pyruvate, 2 mM L-glutamine, 100 mM MEM non-essential amino acids, 5 mM HEPES buffer solution, 55 mM 2-ME, 100 U/ml penicillin, and 100 mg/ml streptomycin; purchased from Gibco (Thermo Fisher Scientific, Scoresby, VIC, Australia)]. Hygromycin B (200 μg/ml) or Geneticin (500 μg/ml) were used as selection markers (Invitrogen) of stable transfectants. A549 cells (ATCC) were maintained in cDMEM [DMEM with 10% heat-inactivated FCS, 2 mM L-glutamine, 1 mM Sodium pyruvate, 100 U/ml penicillin and 100 mg/ml streptomycin; purchased from Gibco].

### Viruses and Infections

Influenza A (A/Switzerland/9715293/2013, A/Hong Kong/4801 /2014, A/California/04/2009, A/PR8) and B (B/Malaysia/2506/04, B/Brisbane/06/2008, B/Massachusetts/2/2012, B/Phuket/3073/ 2013) viruses were grown in the allantoic cavity of day 10-embryonated chicken eggs for 3 days at 35°C and viral titres were determined by plaque assay on MDCK cells. Influenza B viruses were provided by Seqirus, Australia. For virus infections, cells were washed once and resuspended in serum free media before IAV or IBV viruses were added at an MOI of 5. Cells were incubated with virus for 1 h at 37°C, then cells were washed with complete media (10% FCS) and cultured for the appropriate time.

### Antibody Staining and Flow Cytometry

Cells were stained with LIVE/DEAD™ Fixable Near-IR or Aqua (Molecular Probes, Eugene, OR, USA) and a panel of surface markers in MACS buffer (PBS with 0.5% BSA and 2 mM EDTA): HLA-ABC Pe-Cy7 (1:200, w6/32, BD), HLA-ABC AF-700 (1:200, w6/32, BioLegend), HLA-DR BV605 (1:100, L243, BioLegend), CD71 Pe-Cy7 (1:100, RI7217, BioLegend), for 30 min at 4°C. Cells were then washed, fixed and then permeabilized with intracellular antibody staining using the BD Cytofix/Cytoperm kit according to manufacturer's instructions. For intracellular antibody staining the following antibodies were used: HLA-ABC AF-700 (1:200, w6/32, BioLegend), ANP-FITC (1:200, 1331, GeneTex), BNP-FITC (1:200, H89B, ThermoFisher). For the detection of BHA protein on the surface of infected cells, cells were firstly stained with a mouse monoclonal antibody specific for B/Malaysia (MIA1.8E3.1E9) (13) for 30 min at 4°C and then with a secondary goat anti-mouse PE (1:200, BD Pharmigen) for 30 min at 4°C, before fixation with the BD Cytofix/Cytoperm kit according to manufacturer's instructions. Samples were acquired on LSR Fortessa (BD Biosciences). All flow cytometry data were analyzed using FlowJo 10 (FlowJo, LLC).

### Radiolabelling, Immunoprecipitation and Endo-H Sensitivity Analysis

Cells were starved in methionine- and cysteine-free DMEM for 30 min at 37°C and then pulsed in the same media supplemented with ^35^S-labeled methionine and cysteine (Express Protein Labeling Mix, Perkin Elmer) at 200 μCi/ml for 15 min at 37°C. Cells were then washed with ice-cold RF10 and then incubated in RF10 at 37°C. At the selected timepoints, cells were washed in PBS and frozen. Cell pellets were lysed in 0.5% IGEPAL CA-630 (Sigma-Aldrich), 50 mM Tris-HCl (pH 7.4), 5 mM MgCl_2_with Complete Protease Inhibitor Cocktail (Roche), and centrifuged 13,000 × *g* for 10 min to separate nuclei. Lysates were precleared twice with normal mouse serum (Sigma-Aldrich) and protein G–Sepharose and twice with protein G–Sepharose alone. MHC-I was immunoprecipitated using w6/32 antibody and protein G–Sepharose, and the immunoprecipitates were washed in NET buffer (0.5% IGEPAL CA-630, 50 mM Tris-Cl pH 7.4, 150 mM NaCl, 5 mM EDTA) three times. Precipitates were treated with Endoglycosidase Hf (New England Biolabs) according to the manufacturer's instructions. Proteins were denatured in reducing LDS-PAGE sample buffer and separated on NuPAGE 4–12% Bis-Tris precast gels (Life Technologies) before transferring onto PVDF membranes using the iBlot system (Life Technologies). Membranes were dried and exposed to a storage phosphor screen (GE Healthcare) and imaged on a Typhoon imager (GE Healthcare).

### Statistical Analysis

An unpaired Student's *t*-test was used to assess statistical significance in Prism 7 (GraphPad) with ^*^p < 0.05, ^**^p < 0.01, ^***^p < 0.001. Error bars indicate the mean ± SEM.

## Results

### IAV and IBV Downregulate MHC-I in Late Stages of Viral Infection

**Figure 1:**
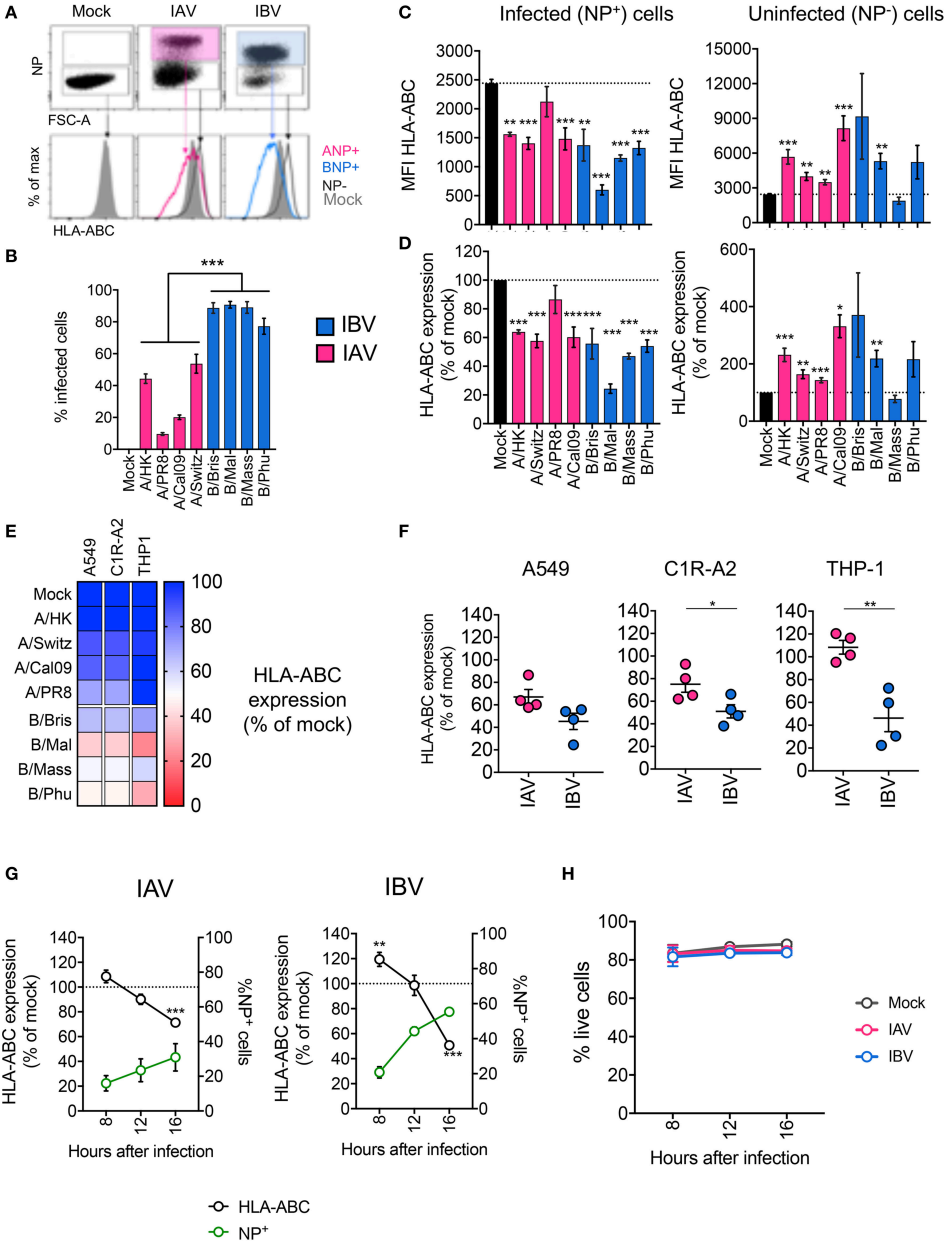
Infection with IAV and IBV leads to downregulation of MHC-I. **(A–C)** Changes in surface MHC-I expression during *in vitro* IAV and IBV infection of A549 cells. Cells were infected with IAV/IBV or mock-treated and MHC-I (HLA-ABC) expression was analyzed on total live mock-treated samples and NP^−^ or NP^+^ for IAV/IBV-treated samples. **(A)** Representative FACS plots of NP staining and MHC-I expression. **(B)** Infections rates for different IAV and IBV strains. **(C)** HLA-ABC expression on uninfected (NP^−^) and infected cells (NP^+^) as geometric Mean Fluorescence Intensities (MFI). **(D)** Relative HLA-ABC expression on uninfected (NP^−^) and infected cells (NP^+^) compared to mock-treated cells. Mean and SEM are shown for *n* = 6, pooled data from two independent experiments, each performed in triplicate. **(E)** Relative HLA-ABC expression on infected cells (NP^+^) compared to mock-treated cells, for a panel of IAV and IBV strains, across cell lines. Mean value shown (*n* = 6, pooled data from two independent experiments, each performed in triplicate). **(F)** Relative HLA-ABC expression on infected cells (NP^+^) compared to mock-treated cells, for IAV and IBV viruses, where each data point represents the mean value (as above) for one strain. **(G)** Timecourse of MHC-I downregulation my IAV and IBV. Relative HLA-ABC expression on infected cells (NP^+^) compared to mock-treated cells, for IAV and IBV. The frequency of NP^+^ cells is also shown. **(H)** Cell viability after influenza infection at different timepoints after infection. Mean and SEM are shown for *n* = 6, pooled data from two independent experiments, each performed in triplicate. Statistical significance was determined using unpaired Student's *t*-test throughout the figure with ^*^p < 0.05, ^**^p < 0.01, ^***^p < 0.001.

To assess whether influenza virus infection affected MHC-I cell surface expression, we infected human alveolar basal epithelial A549 cells, frequently used as an *in vitro* model of influenza virus infection, with a panel of IAV (A/PR8 H1N1, A/Cal09 pdm09H1N1, A/Switzerland A/H3N2, and A/Hong Kong H3N2) and IBV (B/Brisbane Vic, B/Malaysia Vic, B/Massachusetts Yam, B/Phuket Yam) strains. After 16 h of infection, cells were stained for surface MHC-I expression using a pan-MHC-I antibody (w6/32) and intracellularly for IAV- or IBV-derived nucleoprotein (NP) protein (Figure 1A). Using a multiplicity of infection (MOI) of 5, the infection rates, as determined by NP^+^ staining, differed between IAV and IBV (*p* < 0.001) viral strains (Figure 1B). To account for any differences in infection rates, we analyzed MHC-I expression on infected cells (NP^+^) and uninfected cells (NP^−^) in influenza-treated cells, relative to the MHC-I expression on mock-treated cells. For all IAV and IBV strains, with the exception of A/PR8, NP^+^ cells expressed significantly lower (*p* < 0.05) geometric mean fluorescence intensity (gMFI) levels of surface MHC-I as compared to mock treated cells (gMFIs: ~2,400 for mock, ~1,500 for IAV strains, ~600–1,400 for IBV strains) (Figure 1C). In contrast, NP^−^ cells expressed variably higher MFI levels of MHC-I (gMFI: 3,500–9,000) compared to the mock control (Figure 1C, left panel), likely due to the effects of type I IFN secreted by influenza-infected cells (14, 15). Overall, influenza virus infection resulted in ~40% downregulation of surface MHC-I for IAV and ~45–75% loss for IBV (Figure 1D).

To validate these results, we used the same panel of IAV and IBV strains to infect a human class-I reduced (C1R) lymphoblastoid cell line, engineered to stably express the highly prevalent HLA-A2 allele (C1R-A2), as well as the human monocyte-like THP-1 cell line. As above, we normalized for differences between infection rates across cell lines and virus strains, then measured MHC-I expression on infected NP^+^ cells relative to mock-treated cells. Across all three cell lines tested, both IAV and IBV infections resulted in the reduction of MHC-I, although variably so across strains and cell lines (Figure 1E). In A549 cells, the mean percentage of MHC-I expression of the four different strains within IAV or IBV did not differ between IAV and IBV, whereas in C1R-A2 cells, downregulation of MHC-I was significantly greater for IBV (50% MHC-I remaining) than for IAV (75%, *p* = 0.04) (Figure 1F). Additionally, in THP-1 cells, IAV infection did not result in any downregulation of MHC-I (mean MHC-I expression 108% compared to mock-treated cells), while IBV infection resulted in ~56% loss of MHC-I (Figure 1F). The lack of MHC-I downregulation by IAV in THP-1 cells could not be attributed to lower infection rates, as their infection rates were comparable to A549 cells (Figure S1A; Figure 1B).

We next assessed the kinetics of MHC-I downregulation by IAV and IBV in C1R-A2 cells. For both IAVs and IBVs, while infectivity increased over time, MHC-I downregulation was observed at the very late stages of infection (16 h) (Figure 1G). Interestingly, for IBV at 8 h after infection, MHC-I was increased compared to mock-treated cells. The viability of these cells was not compromised following IAV or IBV infection (Figure 1H). Overall, our data show that infection with IAV and IBV results in dynamic changes of MHC-I expression.

### Downregulation of HLA-A, -B, and -C Alleles by IAV and IBV

**Figure 2:**
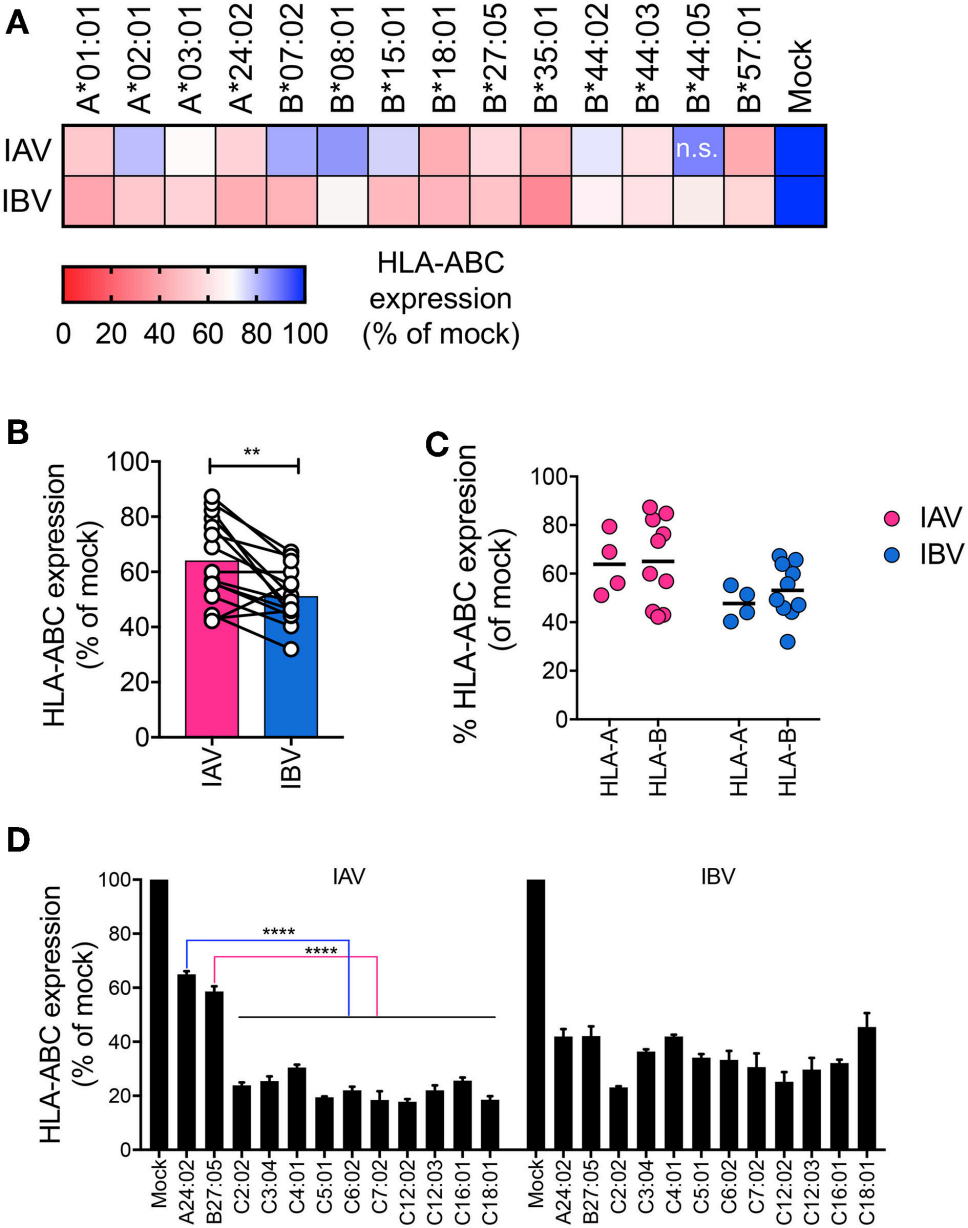
Downregulation of HLA-A, -B, and -C alleles by IAV and IBV. **(A)** Relative HLA-ABC expression on infected cells (NP^+^) compared to mock-treated cells, for IAV and IBV, across C1R lines expressing different HLA-A and -B alleles. Expression after infection for each allele is relative to that allele's respective mock-treated samples. Mean and SEM are shown for *n* = 6, pooled data from two independent experiments, each performed in triplicate. **(B, C)** Relative HLA-ABC expression on infected cells (NP^+^) compared to mock-treated cells, for IAV and IBV, where each data point represents the mean value (as above) for one allele. **(D)** Relative HLA-ABC expression on infected cells (NP^+^) compared to mock-treated cells, for IAV and IBV, across 721.221 lines expressing different HLA-C alleles. A24:02 and B^*^27:05-expressing lines were used for comparison. Expression after infection for each allele is relative to that alleles respective mock-treated samples. Mean and SEM are shown for *n* = 6, pooled data from two independent experiments, each performed in triplicate. Statistical significance was determined using unpaired Student's *t*-test throughout the figure with ^**^p < 0.01, ^****^p < 0.0001.

Downregulation of MHC-I by HIV differs across MHC-I allotypes, whereby, HLA-A allotypes are more susceptible than HLA-B allotypes (16). Since A549 and THP-1 cell lines express a diverse set of HLA-I alleles (A549: A^*^25:01, A^*^30:01, B^*^18, B^*^44:03, C^*^12:03, C^*^16:01, and THP-1: A^*^02, B^*^15, C^*^03) (17, 18), we next assessed how different HLA-A, -B, and C allotypes are downregulated during influenza infection. To that end, we used a library of 14 C1R cell lines transduced with single different HLA-A (A^*^01:01, A^*^02:01, A^*^03:01, A^*^24:02) or HLA-B (B^*^07:02, B^*^08:01, B^*^15:01, B^*^18:01, B^*^27:05, B^*^35:01, B^*^44:02, B^*^44:03, B^*^44:05, B^*^57:01) alleles. These cells were infected with a representative IAV (A/Switzerland H3N2) or IBV strain (B/Malaysia-Vic) (Figure S1B) and MHC-I expression was assessed at 16 h after infection. For all allotypes, MHC-I was significantly downregulated in NP^+^ cells following IAV infection (16–58% reduction) and IBV infection (37–69% reduction), as compared to their respective mock-treated cells (*p* < 0.05), with the exception of HLA-B^*^44:05 during IAV infection (Figure 2A). In general, when all the HLA alleles were grouped together, IBV infection resulted in significantly greater downregulation of MHC-I (*p* = 0.002, *n* = 14 alleles) (Figure 2B). However, there were no differences in MHC-I downregulation between HLA-A and HLA-B alleles for either IAV or IBV (Figure 2C).

To assess downregulation of HLA-C alleles, we used a library of human lymphoblastoid HLA-null 721.221 cell lines, transduced with 10 different HLA-C alleles (C^*^02:02, C^*^03:04, C^*^04:01, C^*^05:01, C^*^06:02, C^*^07:02, C^*^12:02, C^*^12:03, C^*^16:01, C^*^18:01). 721.221 cells expressing A^*^24:02 or B^*^27:05 were used for comparison between HLA-A, -B, and -C alleles in the same cell type. All HLA-C alleles tested were significantly (*p* < 0.0001) downregulated by either IAV or IBV, with only ~20–40% of surface MHC-I remaining after 16 h of infection (Figure 2D, Figure S1C). Interestingly, during IAV infection, downregulation of all HLA-C alleles (70–80% reduction) was significantly >A^*^24:02 (36% reduction) or B^*^27:05 (42% reduction) (*p* < 0.0001). Overall, downregulation of MHC-I by IAV and IBV viruses occurs across a range of HLA-A, B-, and C- allotypes.

### Differential Mechanisms Drive Downregulation of MHC-I by IAV and IBV

**Figure 3:**
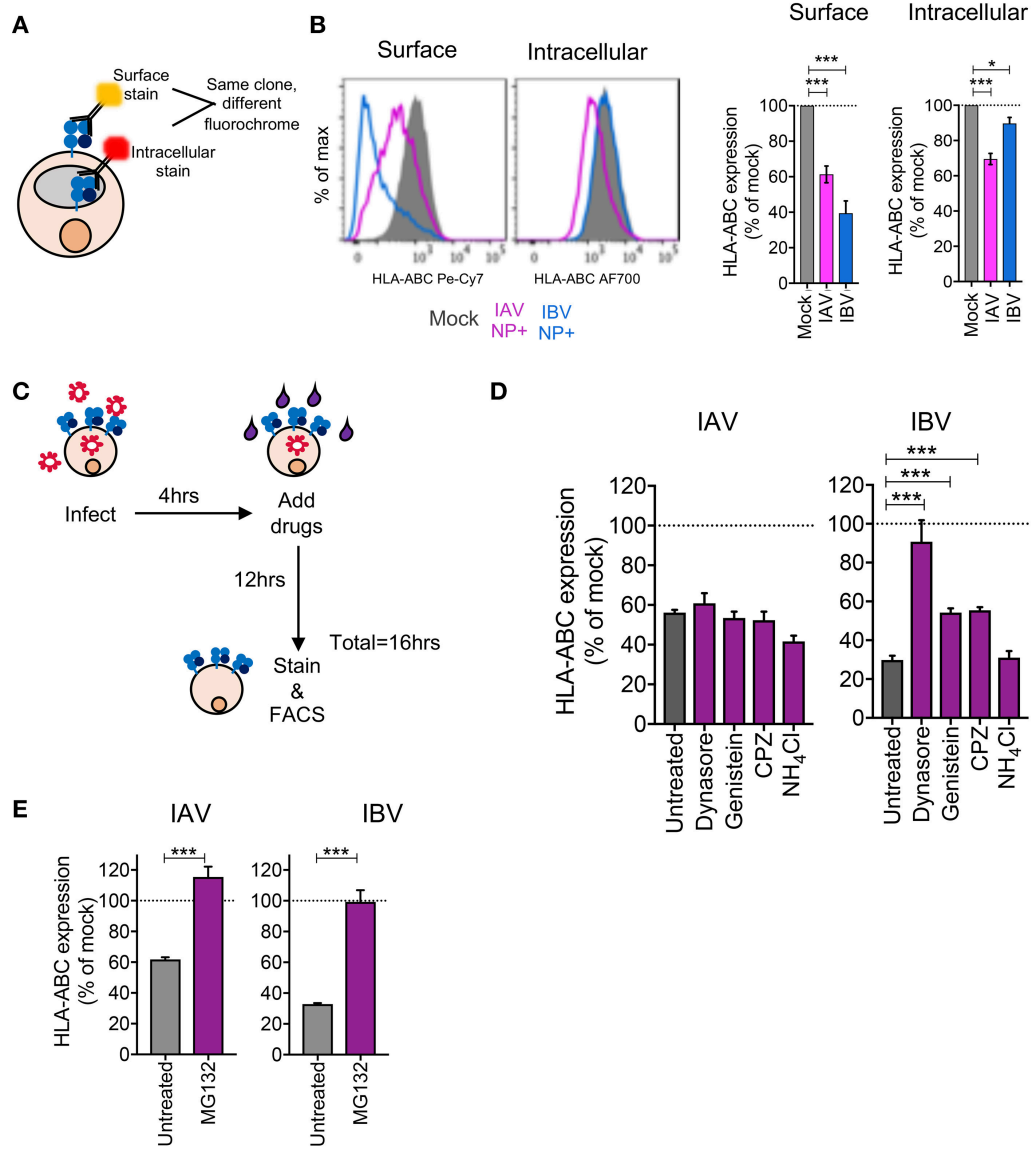
Differential mechanisms drive downregulation of MHC-I by IAV and IBV. **(A, B)** Downregulation of surface vs. intracellular HLA-ABC during IAV and IBV infection. **(A)** Outline of experimental design. **(B)** Representative FACS plots and bar graphs for surface and intracellular HLA-ABC expression, for total live cells in mock-treated samples and NP^+^ cells for IAV/IBV-treated samples. **(C–E)** Drug-mediated rescue of HLA-ABC downregulation. **(C)** Outline of experimental design. **(D)** Relative HLA-ABC expression on infected cells (NP^+^) compared to mock-infected untreated cells, for IAV and IBV, treated with inhibitors of endocytosis and lysosomal degradation. **(E)** Relative HLA-ABC expression on infected cells (NP^+^) compared to mock-infected untreated cells, for IAV and IBV, treated with inhibitors of the proteasome. Mean and SEM are shown, throughout the figure, for *n* = 6, pooled data from two independent experiments, each performed in triplicate. Statistical significance was determined using unpaired Student's *t*-test throughout the figure with ^*^p < 0.05, ^***^p < 0.001.

Having observed substantial MHC-I downregulation across virus strains, cell lines, and HLA allotypes, we dissected the mechanisms driving MHC-I downregulation during IAV and IBV infections. Firstly, to assess whether downregulation of MHC-I was specific to surface MHC-I or whether it was depleted intracellularly, C1R-A2 cells were infected with IAV (A/Switzerland H3N2) or IBV (B/Malaysia-Vic) viruses before surface and intracellular MHC-I expression were measured using the same anti-MHC-I antibody (w6/32) conjugated to two different fluorochromes (Pe-Cy7 for surface staining and AF-700 for intracellular staining (Figure 3A). Interestingly, following IAV infection, NP^+^ cells had significantly reduced levels (*p* < 0.001) of both surface (61% of mock) and intracellular MHC-I (70% of mock) (Figure 3B). In contrast, following IBV infection, intracellular MHC-I levels in NP^+^ cells were only marginally affected (90% of mock, *p* = 0.01), while surface levels were significantly reduced (*p* < 0.001, 39% of mock) (Figure 3B). This discrepancy between IAV and IBV viruses suggested that the two influenza types may employ different mechanisms toward MHC-I downregulation.

We subsequently used drug inhibitors targeting different pathways of endocytosis and recycling (Table 1), whereby C1R-A2 cells were infected with IAV or IBV for 4 h before drug was added for the remaining 12 h of infection (Figure 3C). Treatment of uninfected cells with endocytosis inhibitors, only had a modest effect on MHC-I expression (Figure S2A). In IAV-infected NP^+^ cells, there was a ~44% reduction in MHC-I cell surface expression in the untreated group, and this remained unchanged when drugs were added to block endocytosis (Figure 3D). In contrast, while untreated IBV-infected NP^+^ cells had a ~70% decrease in their surface MHC-I, treatment with Dynasore, an inhibitor targeting dynamin-dependent endocytosis, resulted in a significantly higher surface MHC-I expression (~10% loss of mock-treated cells, *p* = 0.003) (Figure 3D). Treatment with Genistein, which inhibits clathrin-independent endocytosis, or Chlorpromazine (CPZ, inhibits clathrin-dependent endocytosis) also significantly reduced downregulation (*p* < 0.001), although partially (~55% reduction of MHC-I expression as compared to ~75% in mock-treated cells) (Figure 3D). Treatment with NH_4_Cl, which blocks lysosomal degradation, did not have a significant effect on MHC-I downregulation.

**Table 1:** Summary of drug inhibitors, their target pathway and their effect on MHC-I downregulation.

**Inhibitor**	**Concentration**	**Pathway targeted**	**MHC-I recovery during IAV infection**	**MHC-I recovery during IBV infection**
Dynasore	50 μM	Dynamin-dependent endocytosis	**−**	+++
Genistein	25 μg/ml	Clathrin-independent endocytosis	–	+
Chlorpromazine (CPZ)	10 μg/ml	Clathrin-dependent endocytosis	–	+
NH_4_Cl	10 μM	Lysosome-mediated degradation	–	–
MG132	5 μM	Proteosome-mediated degradation	+++	++

To further dissect the mechanism of MHC-I downregulation, particularly following IAV infection, we treated IAV- or IBV-infected cells with the proteasome inhibitor MG132, as above, to determine whether preventing proteasome-mediated degradation could prevent or restore MHC-I downregulation. Indeed, treatment of either IAV- or IBV-infected cells with MG132 significantly rescued MHC-I downregulation to ~100% (*p* < 0.001, Figure 3E).

In terms of the infection rates, it should be noted that a treatment with the endocytosis inhibitors resulted in a modest but significant reduction (*p* < 0.05) in the frequency of ANP^+^ and BNP^+^ cells Figure S2B), although this did not affect the gMFI expression levels of ANP and BNP proteins within NP^+^ cells (*p* > 0.05), with the exception of a modest decrease in BNP expression following treatment with Chlorpromazine (Figure S2C). Similarly, the viability of cells treated with the endocytosis inhibitors following influenza virus infection was not significantly different from that of infected and untreated cells, again with the exception of Chlorpromazine (Figure S2D). In case of MG132, however, both IAV and IBV infection rates (37 and 35%, respectively, with MG132) were significantly reduced (*p* < 0.01) compared to untreated cells (55 and 70%, respectively, in the untreated groups) (Figure S2E), and the BNP gMFI expression levels was significantly (*p* < 0.001) reduced from 5,312 untreated to 2,243 with MG132 (Figure S2F). Additionally, cell viability was substantially lower following MG132 treatment (~30% compared to 80–90% for untreated cells) (Figure S2G), which is likely due to the ability of MG132 to induce cell apoptosis (19). Thus, while our flow cytometric analysis is focused on live cells that express influenza NP proteins, the effects of MG132 on rescuing IBV-mediated downregulation of MHC-I, may be a non-specific result of reduced infection/viral protein expression.

Overall, our data suggest a mechanism of MHC-I downregulation whereby IAV causes loss of total MHC-I from the cell surface and intracellularly that is independent of endocytosis but dependent on proteasomal degradation, while IBV causes a loss of surface MHC-I, but not intracellular MHC-I, which can be prevented or reversed by blocking endocytosis of surface MHC-I.

### Delayed Trafficking of MHC-I to the Cell Surface During IBV Infection

Having shown that IAV infection results in a global loss of MHC-I, likely in a proteasome mediated manner, we focused on dissecting the mechanism underlying the loss of surface MHC-I by IBV. Given that IBV causes MHC-I downregulation on the cell surface, but not intracellularly, and that this can be rescued by inhibiting endocytosis, we investigated whether the loss of MHC-I from the cell surface following IBV infection was due to: (1) delayed MHC-I trafficking to the cell surface; or (2) whether IBV infection increased MHC-I internalization and degradation rate.

**Figure 4:**
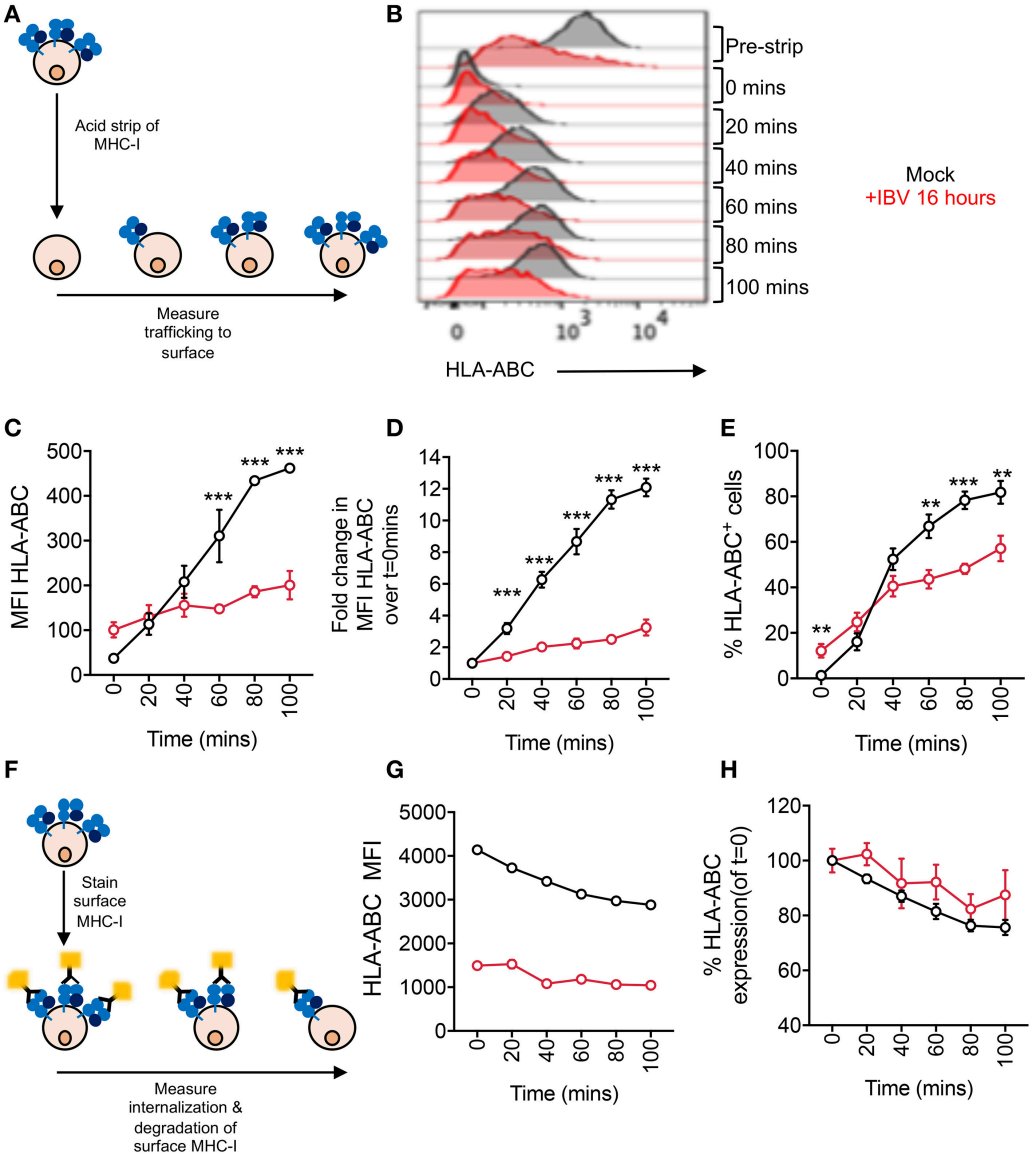
Delayed trafficking of MHC-I to the cell surface during IBV infection. **(A– E)** Rates of MHC-I trafficking to the cell surface during IBV infection. **(A)** Outline of experimental design. **(B)** Representative FACS plots for mock and IBV-treated cells. **(C)** MHC-I expression at different timepoints after stripping, expressed as MFIs. **(D)** Fold change in MHC-I expression at different timepoints after stripping, relative to 0 min after stripping. **(E)** Frequency of MHC-I^+^ cells at different timepoints after stripping. **(F–H)** Rates of MHC-I internalization and degradation from the cell surface during IBV infection. **(F)** Outline of experimental design. **(G)** MHC-I expression at different timepoints after staining, expressed as MFIs. **(H)** Relative MHC-I expression at different timepoints after staining, relative to 0 min. **(C,G)** Mean and SEM are shown, *n* = 3, from one experiment, due to variations in flow cytometer settings between experiments, with similar trends observed between two independent experiments. **(D,E,H)** Mean and SEM are shown, for *n* = 6, pooled data from two independent experiments, each performed in triplicate. Statistical significance was determined using unpaired Student's *t*-test throughout the figure with ^**^p < 0.01, ^***^p < 0.001.

To assess the rate at which MHC-I traffics to the cell surface, we exposed mock or IBV-infected C1R-A2 cells to an acid buffer (pH 2.0), which results in the loss of the MHC-I complex from the cell surface (“acid-stripping”) (20). We then measured the increase of surface MHC-I expression at 20 min intervals for up to 100 min (Figure 4A). Surface MHC-I expression of mock-infected cells was significantly higher (*p* < 0.001) compared to IBV-infected cells at 60 min after acid-stripping and continued to be significantly higher up to 100 min (Figures 4B,C). Overall, MHC-I expression on mock-infected cells gradually increased up to 12-fold at 100 min after acid-stripping, when compared to 0 min. In contrast, IBV-infected cells (NP^+^) only increased by 2-fold after 100 min (Figure 4D). Consistently, the frequency of cells expressing MHC-I increased from 1 to 80% for mock-infected cells, but only from 12 to 57% for IBV-infected cells, with significant differences (*p* < 0.001) in the mock-infected cells from 60 min onwards after stripping (Figure 4E). These data indicate that during IBV infection, the rate at which MHC-I traffics to the cell surface is significantly reduced.

To determine whether the rate of surface MHC-I internalization and subsequent degradation are also affected, we fluorescently labeled surface MHC-I of mock- or IBV-infected cells (16 h) and, after washing off any excess antibody, determined the rate at which the MHC-I signal was reduced, reflecting internalization and degradation of MHC-I (Figure 4F). Both mock and IBV-infected cells exhibited a slight gradual loss of MHC-I although this was less evident for IBV due to already significantly reduced MHC-I levels at 16 h after infection (Figure 4G). Relative to their respective *t* = 0, both mock and IBV-infected cells had a small reduction (~20%) in MHC-I expression by 100 min after staining (Figure 4H), suggesting that IBV does not increase the rate at which MHC-I is internalized and degraded. While this may seem contrary to the ability of the endocytosis inhibitor Dynasore to rescue or prevent IBV-mediated MHC-I downregulation, it should be noted that Dynasore may be retaining existing MHC-I on the cell surface thus stabilizing its levels, regardless of whether new MHC-I is delivered to the surface or not.

Thus, during IBV infection, MHC-I trafficking to the cell surface is delayed, suggesting that MHC-I is retained in an intracellular compartment during IBV infection.

### MHC-I Is not Retained in the ER During IBV Infection

**Figure 5:**
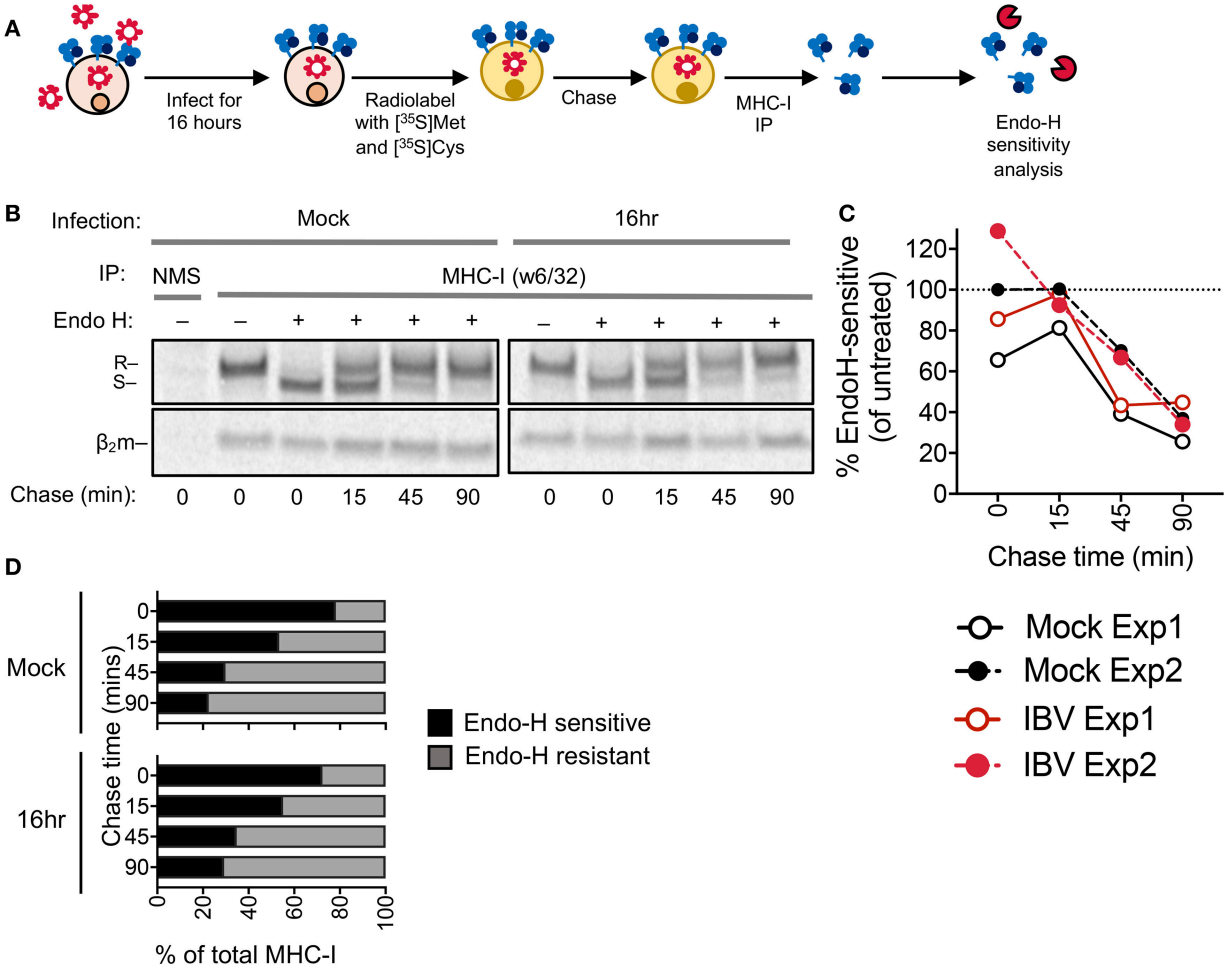
MHC-I is not retained in the ER during IBV infection. **(A)** Outline of experimental design. **(B)** Autoradiograms of Endo-H sensitivity of immunoprecipitated (IP) MHC-I (w6/32) from C1R-A2 cells, which were either infected with IBV for 16 h or mock-treated, and then metabolically radiolabelled with [^35^S]methionine and [^35^S]cysteine and chased for the indicated timepoints. NMS: immunoprecipitated obtained with normal mouse serum as a control. R: Endo-H resistant MHC-I. S: Endo-H sensitive MHC-I. **(C)** Percent of Endo-H sensitive MHC-I in mock or IBV-treated cells, based on “R” band intensity at each timepoint compare to Endo-H untreated band intensity (columns 2 or 7). **(D)** Fractions of Endo-H sensitive/resistant MHC-I in mock or IBV-infected cells, based on S/R band intensity relative to their sum for each timepoint**. (B–D)** Data are representative of two independent experiments.

Retention of MHC-I within the ER is a common immune evasion strategy employed by many viruses, such as human cytomegalovirus (21) and adenovirus (22). To determine whether IBV retains MHC-I in the ER, we performed an Endo-H sensitivity analysis on IBV-infected (16 h) or mock-treated C1R-A2 cells to measure MHC-I in the ER (Endo-H sensitive) or in the Golgi and post-Golgi compartments (Endo-H resistant) over 90 min following MHC-I radiolabelling and immunoprecipitation (Figure 5A). For both mock-treated and IBV-infection, at time 0 after radio-labeling the majority of radiolabelled MHC-I were Endo-H sensitive, indicative of ER localization (Figures 5B–D), whereas only 20–30% of newly synthesized MHC-I were Endo-H resistant, indicative of passage through the Golgi. However, this gradually increased to 70–80% by 90 min (Figures 5B–D). Therefore, the kinetics of Endo-H resistance were comparable between IBV-infected and mock-treated cells (Figures 5B–D), suggesting that MHC-I is not retained in the ER and traffics with normal kinetics through the Golgi apparatus during IBV infection.

### Selective Downregulation of Surface Host but not Viral Surface Proteins During IBV Infection

**Figure 6:**
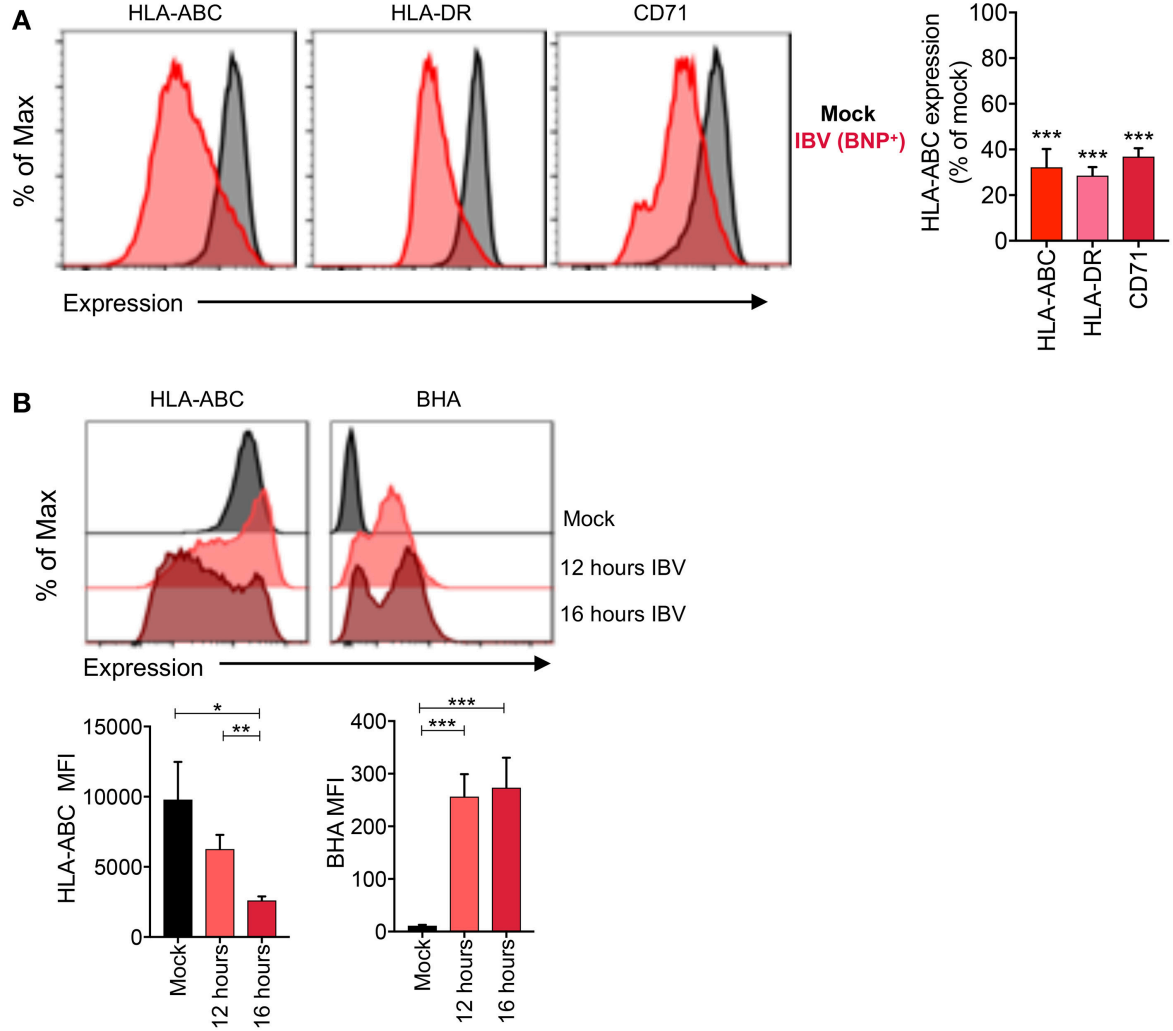
Selective loss of host but not viral surface proteins during IBV infection. **(A)** Expression levels of host (MHC-I, HLA-DR, CD71) proteins and the IBV HA protein on C1R-A2 cells infected with IBV for 16 h or mock-treated. **(B)** No downregulation of the BHA protein between 12 and 16 h. Representative FACS plots are shown. Mean and SEM are shown, throughout the figure, for *n* = 6, pooled data from two independent experiments, each performed in triplicate. Statistical significance was determined using unpaired Student's *t*-test throughout the figure with ^*^p < 0.05, ^**^p < 0.01, ^***^p < 0.001.

To determine whether downregulation is specific to MHC-I or whether it is also applicable to other surface proteins, we assessed expression of MHC-II (HLA-DR) and the transferrin receptor (TfR or CD71) on IBV-infected and mock-treated cells. All three surface proteins (MHC-I, HLA-DR, and CD71), showed significant downregulation (*p* < 0.0001) on IBV NP^+^ cells, with each protein having a ~70% reduction in expression levels (Figure 6A).

The downregulation of surface proteins by IBV in the late stages of infection is intriguing, as altered protein trafficking to the cell surface would need to be selective to host, but not viral proteins, so as not to interfere with virion assembly. To determine whether this might be the case, we measured the surface expression levels of host proteins (MHC-I) and the viral IBV haemagglutinin (BHA) protein at 12 and 16 h after infection. Consistent with our previous data, IBV infection resulted in significant downregulation of MHC-I protein at 16 h after infection compared to mock-treated cells (*p* = 0.02) (Figure 6B). Importantly, the surface expression of MHC-I was significantly reduced between 12 and 16 h (*p* = 0.006). However, the surface expression of the BHA protein was stable between 12 and 16 h [MFI of 257 and 273, respectively (*p* = 0.82)] (Figure 6B), suggesting that the block on trafficking imposed by IBV does not affect the trafficking of viral proteins to the cell surface.

Overall, our data show that infection with IAVs and IBVs leads to downregulation of MHC-I in the late stages of infection via two distinct mechanisms. While IAV causes a global loss of MHC-I within influenza-infected cells, IBV infection results in the preferential loss of MHC-I molecules from the cell surface, due to delayed MHC-I trafficking to the cell surface, resulting from retaining MHC-I intracellularly during IBV infection.

## Discussion

Given that IAV and IBV circulate annually and current influenza virus vaccines only provide modest efficacy (2), a better understanding of the immune response to IAV and IBV and the underlying host-pathogen interactions are necessary to improve vaccine design. Due to the ability of CD8^+^ T cells to confer broad and universal cross-protection, a deeper understanding of T cell responses to IAV and, even more so the understudied but clinically relevant IBV, is needed. Here, we show that both influenza A and B viruses downregulate MHC-I in the late stages of their infection cycles *in vitro*, albeit via two different mechanisms. This observation could have significant immunological consequences.

MHC-I downregulation observed after influenza virus infection is specific for IAV- or IBV-infected cells, as determined by NP intracellular expression, while uninfected “bystander” cells exhibited an increase in MHC-I expression, although variably across influenza strains. This could reflect the activity of IFN released by infected cells as IFN can cause upregulation of MHC-I (14). While this downregulation was only modest for IAV (30–40% reduction of MHC-I), it was substantially more pronounced for IBV (60–70% reduction), and this was consistent across multiple IAV/IBV strains, cell lines, and HLA alleles. The kinetics of MHC-I downregulation are intriguing as this only occurs in the late stages of infection (16 h), when the influenza virions assemble and bud off. It would also be important to verify our observation of MHC-I downregulation by IAV and IBV in primary cells as well as *in vivo* models of influenza infection.

Our data are suggestive of two different mechanisms of downregulation by IAV and IBV. We hypothesize that IAV leads to either reduced transcription of MHC-I, specifically or as a result of general host translation shut-off, or degradation of intracellular pools of MHC-I, which is supported by the effects of proteasomal inhibitor MG132 on IAV-mediated downregulation and the loss of intracellular MHC-I. Interestingly, it has been previously reported that in the late stages of infection by IAV (16 h) the mRNA levels of MHC-I, among other antiviral transcripts, are greatly reduced (14). This is consistent with the ability of IAV to induce host protein shut-off in multiple ways (23). We hypothesize that this effect may be driving the reduced expression of MHC-I.

In contrast, we postulate that during IBV infection MHC-I is retained in an undefined intracellular location. Our hypothesis that IBV retains MHC-I, and likely other host proteins, in an intracellular compartment is supported by the delayed trafficking of MHC-I to the cell surface, the ability of endocytosis inhibitors to restore or prevent this process, contrary to that of IAV. Investigations into IBV-mediated MHC-I downregulation using radiolabelling and an Endo-H sensitivity assay showed that the kinetics MHC-I traffic through the Golgi were comparable between mock-treated and IBV-infected cells, supporting the idea that the retention in MHC-I occurs after trafficking though the Golgi apparatus, where protein glycosylation renders molecules Endo-H resistant. Considering MHC-I had reduced arrival at the cell surface in IBV infected cells, this suggests that the MHC-I molecules are retained en route to the plasma membrane and potentially sequestered in an undefined vesicular location. The exact location of the MHC-I-containing vesicle remains elusive, and further studies are underway to assess that. In addition, identifying the viral factors driving this downregulation of MHC-I in the late stages of IAV and IBV would be of utmost importance in dissecting the mechanisms and immunological consequences of MHC-I downregulation, as well as the differences between IAV and IBV.

Downregulation of MHC-I often leads to reduced CD8^+^ T cell responses (12). While we have yet to determine the biological consequences of the observed downregulation, we speculate that there might be differences between IAV and IBV, strains and cell types. Particularly, since ~60–70% of MHC-I remains on the cell surface of IAV-infected cells, the effects on the CD8^+^ T cell response may only be modest, especially for CD8^+^ T cells of high functional avidity. It is also pertinent to note that since downregulation is only observed in the late stages of infection, CD8^+^ T cells would still be able to recognize infected cells prior to that. In fact, at 8 h after IBV infection, MHC-I is higher compared to mock-treated cells, which may increase the susceptibility of these infected cells to CD8^+^ T cell-mediated killing. Additionally, downregulation of MHC-I would likely make infected cells more susceptible to NK cell-mediated cytotoxicity, especially in the late stages of infection, when viral proteins are abundant on the cell surface which can be targeted by antibodies to initiate antibody-dependent cellular cytotoxicity.

The effect of IBV appears not to be specific for MHC-I, as CD71 (TfR) and HLA-DR (MHC-II) were also downregulated from the surface of infected cells. Combined with the timing of this, it is conceivable that the virus manipulates host intracellular trafficking to promote the delivery of viral proteins to the cell surface for virion assembly and egress. Indeed, the cell surface expression levels of the BHA protein were stable between 12 and 16 h. Thus, this process may have not specifically evolved to evade CD8^+^ T cells but MHC-I downregulation may be a side-effect of the IBV life cycle.

Overall, our study suggests that in the late stages of infection, MHC-I levels are significantly reduced on both IAV- and IBV- infected cells via two different processes, likely as a by-product of the viral life cycle. This downregulation could have significant effects on the immune response against IAV and IBV and also highlights biological differences between IAV and IBV.

## Author Contributions

MK, TN, and KK designed the research. MK, HM, TA, SF, PI, and NM performed the experiments. MK, HM, TN, and KK analyzed data. HM, SR, PR, AP, JMc, JMi, AB, and JV made significant contributions to reagents or experimental design. MK, TN, and KK wrote the manuscript. All authors read and approved the manuscript.

### Conflict of Interest Statement

SR is an employee of Seqirus Ltd. and has no conflict of interest in the material presented. The remaining authors declare that the research was conducted in the absence of any commercial or financial relationships that could be construed as a potential conflict of interest.

## Supplementary Material

Figure S1 Infection rates of THP-1 cells and C1R or 221 cells expressing different HLA allotypes. **(A)** Infections rates for THP-1 cells infected with different IAV or IBV strains. **(B)** Infections rates for different HLA expressing C1R cells infected with IAV or IBV. **(C**) Infections rates for different HLA expressing 221 cells infected with IAV or IBV. Mean and SEM are shown, throughout the figure, for *n* = 6, pooled data from two independent experiments, each performed in triplicate.

Figure S2 Effect of drug inhibitors on infection rates, NP expression levels and cell viability. **(A)** Effect of drug treatment on MHC-I expression in uninfected cells. **(B,C)** Infections rates (%NP^+^) **(A)** and NP expression within NP^+^ cells **(B)** for cells infected with IAV or IBV and treated with endocytosis inhibitors. **(D)** Cell viability for cells infected with IAV or IBV and treated with endocytosis inhibitors. Mock cells were not infected and untreated. **(E,F)** Infections rates (%NP^+^) **(E)** and NP expression within NP^+^ cells **(F)** for cells infected with IAV or IBV and treated with MG132. **(G)** Cell viability for cells infected with IAV or IBV and treated with endocytosis inhibitors. Mock cells were not infected and untreated. Mean and SEM are shown, throughout the figure, for *n* = 6, pooled data from two independent experiments, each performed in triplicate. Statistical significance was determined using unpaired Student's *t*-test throughout the figure with ^*^*p* < 0.05, ^**^*p* < 0.01, ^***^*p* < 0.001.
