# Supplementary figures and images for: Downregulation of MHC Class I Expression by Influenza A and B Viruses
Source: Front Immunol. 2019 May 29;10:1158. doi: 10.3389/fimmu.2019.01158 (PMC6548845; doi:10.3389/fimmu.2019.01158)

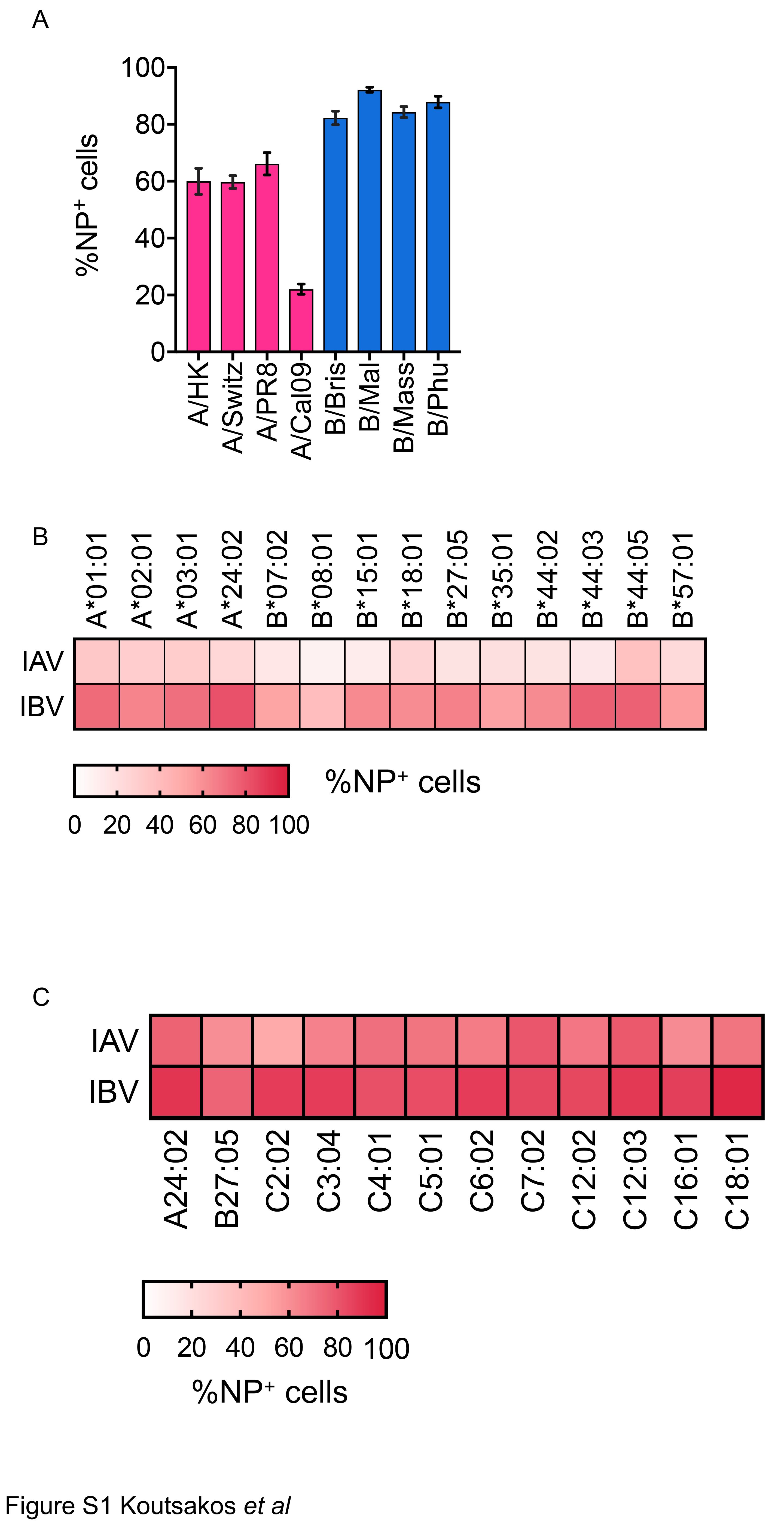

Supplement: Figure S1 — Infection rates of THP-1 cells and C1R or 221 cells expressing different HLA allotypes. (A) Infections rates for THP-1 cells infected with different IAV or IBV strains. (B) Infections rates for different HLA expressing C1R cells infected with IAV or IBV. (C) Infections rates for different HLA expressing 221 cells infected with IAV or IBV. Mean and SEM are shown, throughout the figure, for n = 6, pooled data from two independent experiments, each performed in triplicate. [file Image_1.TIF]

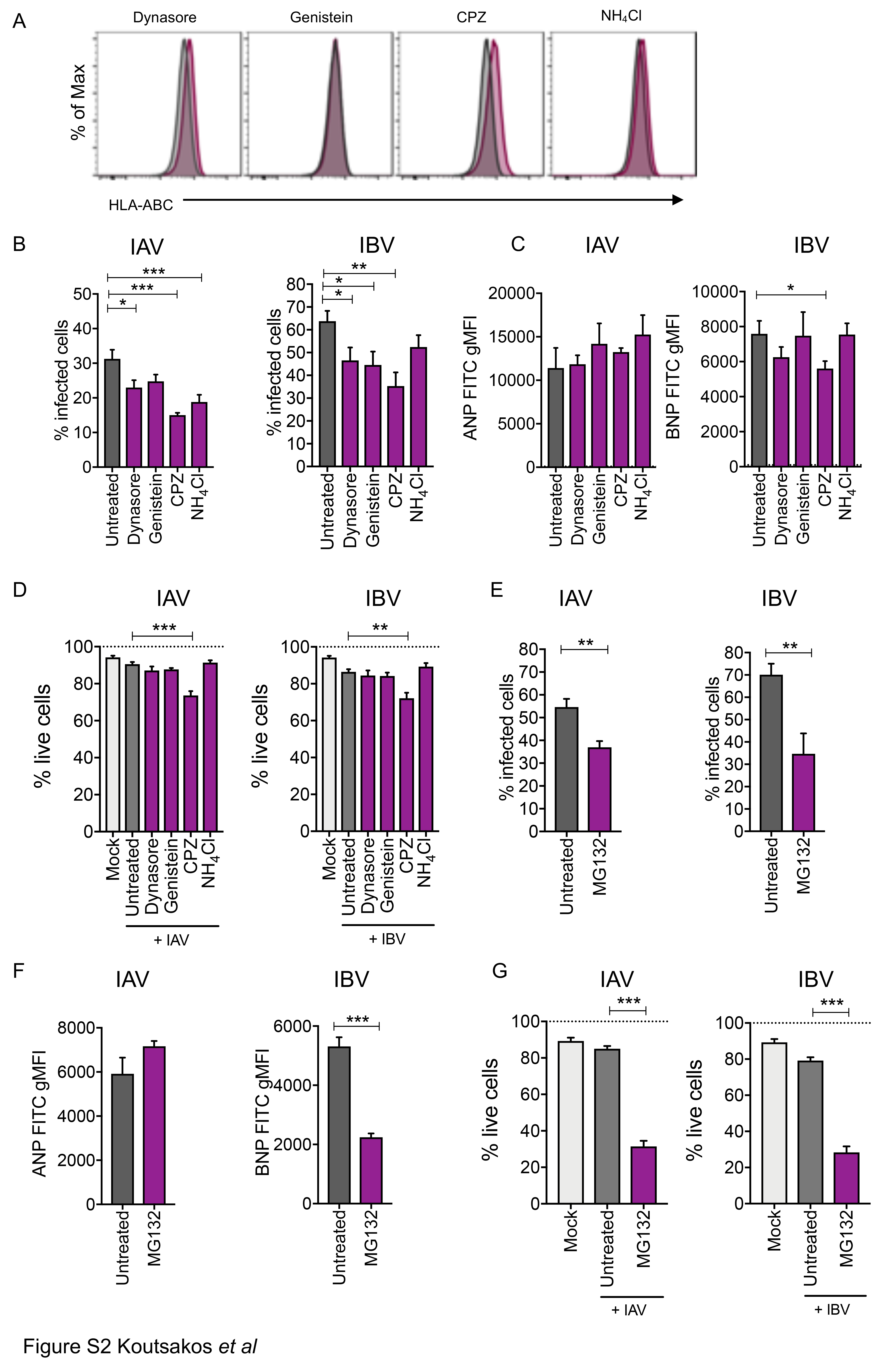

Supplement: Figure S2 — Effect of drug inhibitors on infection rates, NP expression levels and cell viability. (A) Effect of drug treatment on MHC-I expression in uninfected cells. (B,C) Infections rates (%NP+) (A) and NP expression within NP+ cells (B) for cells infected with IAV or IBV and treated with endocytosis inhibitors. (D) Cell viability for cells infected with IAV or IBV and treated with endocytosis inhibitors. Mock cells were not infected and untreated. (E,F) Infections rates (%NP+) (E) and NP expression within NP+ cells (F) for cells infected with IAV or IBV and treated with MG132. (G) Cell viability for cells infected with IAV or IBV and treated with endocytosis inhibitors. Mock cells were not infected and untreated. Mean and SEM are shown, throughout the figure, for n = 6, pooled data from two independent experiments, each performed in triplicate. Statistical significance was determined using unpaired Student's t-test throughout the figure with *p < 0.05, **p < 0.01, ***p < 0.001. [file Image_2.TIF]
